# Integrating Inflammasome Signaling in Sexually Transmitted Infections

**DOI:** 10.1016/j.it.2016.08.004

**Published:** 2016-10

**Authors:** Christopher Lupfer, Paras K. Anand

**Affiliations:** 1 Department of Biology, Missouri State University, Springfield, MO 65809, USA; 2 Infectious Diseases and Immunity, Imperial College London, London W12 0NN, UK

**Keywords:** NLRP3, NLRC4, IFI16, AIM2, caspase-1, inflammasome, IL-1β, IL-18, cancer, oncogenic, tumor, immunopathology, inflammation, sexually transmitted infections

## Abstract

Inflammasomes are cytosolic multiprotein platforms with pivotal roles in infectious diseases. Activation of inflammasomes results in proinflammatory cytokine signaling and pyroptosis. Sexually transmitted infections (STIs) are a major health problem worldwide, yet few studies have probed the impact of inflammasome signaling during these infections. Due to the dearth of appropriate infection models, our current understanding of inflammasomes in STIs is mostly drawn from results obtained *in vitro*, from distant infection sites, or from related microbial strains that are not sexually transmitted. Understanding how inflammasomes influence the outcome of STIs may lead to the development of novel and effective strategies to control disease and prevent transmission. Here we discuss and highlight the recent progress in this field.

## Trends

Distinct inflammasomes can be activated during sexually transmitted infections (STIs), often in discrete cell compartments. The crosstalk between multiple inflammasome sensors is central to maintaining tissue homeostasis.

Contrary to popular belief, inflammasome activation can generate detrimental inflammatory responses during STIs *in vivo*. The outcome of inflammasome activity is governed by the complex host–pathogen interaction in the infected tissue.

Inflammasome effector molecules can yield disparate functional consequences in different tissues, suggesting the need to employ appropriate infection models.

Ligand internalization is not a necessary prerequisite for cytosolic NLRP3 activation. Engagement of certain plasma membrane-localized receptors by sexually transmitted pathogens or their ligands elicits both priming and activation signals for the NLRP3 inflammasome.

## Inflammasomes

Inflammasomes are multiprotein complexes activated in response to various pathogen-associated molecular patterns (PAMPs), such as bacterial lipopolysaccharide (LPS) and flagellin, or in response to cellular damage-associated molecular patterns (DAMPs), including extracellular ATP and fluctuations in cytoplasmic ion concentrations 1, 2, 3. Detection of PAMPs or DAMPs can occur via one of the cytoplasmic pattern recognition receptors of the NOD-like receptor (NLR) or AIM2-like receptor (ALR) (also known as Pyhin proteins) family. These include, but may not be limited to, NLRP1, NLRP3, NLRC4, AIM2, and IFI16 [4]. Following activation, the NLR or ALR interacts with the adaptor protein apoptosis-associated speck-like protein containing a CARD (ASC), which then recruits caspase-1 into the complex. Under certain conditions, caspase-8 is also recruited into the inflammasome complex 5, 6. Activation of caspase-1 in the inflammasome results in the proteolytic cleavage and activation of two important proinflammatory cytokines, IL-1β and IL-18. Furthermore, inflammasome activation results in the cleavage and activation of gasdermin D, which induces a proinflammatory form of cell death known as pyroptosis 7, 8. Caspase-11 in mice (the ortholog of human caspase-4/5) is also involved in NLRP3-mediated inflammasome activation in response to certain Gram-negative bacterial infections 5, 9, 10, 11. NLRP3 inflammasome activation dependent on caspase-11 is termed the noncanonical NLRP3 inflammasome and is initiated by cytoplasmic LPS that activates caspase-11, which in turn activates gasdermin D and facilitates NLRP3-mediated activation of caspase-1 7, 8, 9. Importantly, caspase-11 can induce pyroptosis, but not IL-1β maturation, independently of caspase-1. [12]. Some inflammasomes, such as NLRP1b and NAIP/NLRC4, are directly activated by bacterial PAMPs 13, 14, 15. Other inflammasomes, like NLRP3 and, under certain conditions, AIM2 require a priming step where their expression is increased through activation of other signaling pathways such as Toll-like receptor (TLR) or type I interferon (IFN-α/β) signaling. This in turn activates the NF-κB, IRF-1, or STAT1/2 transcription factors to increase the expression of NLRP3 and AIM2 as well as guanylate-binding proteins (GBPs), caspase-11, pro-IL-1β, and pro-IL-18 9, 16, 17, 18.

STIs are a major health problem worldwide. Despite extensive efforts and recent awareness, only limited success has been achieved in defying STIs. The World Health Organization (WHO) estimates that more than 1 million new STIs occur every day globally. In the USA alone, there are 110 million total STIs and 20 million new infections are acquired each year, with young people being particularly at risk and costing tens of billions of dollars in treatment 19, 20. Although exact statistics are unavailable, it is believed that there is a significantly higher burden in less-developed countries where the social stigma associated with STIs results in underreporting. Inflammasomes have emerged as critical hubs of innate immunity in infectious diseases, yet only a limited number of studies have investigated the impact of inflammasome signaling in STIs. It is becoming increasingly clear that innate immunity and inflammasomes mediate important functions in the genital tract (Box 1). Table 1 lists the inflammasome receptors and their known activation signals during STIs. However, lack of appropriate models that employ vaginal or other relevant *in vivo* infection routes limits our understanding of the importance of inflammasomes during STIs. Furthermore, several pathogens that cause STIs are restricted to humans and the development of validated surrogates or new infection models is needed. Below we review the current state of understanding of inflammasome signaling during STIs with emphasis on the involvement of the inflammasome in animal models or clinical samples.

## Mechanisms of Inflammasome Activation in STIs

Understanding the mechanism by which pathogens activate the inflammasome not only enhances our basic understanding of the mechanisms of disease but also provides useful insight into potential therapeutic strategies. *Candida albicans* is the major fungal species known to cause STI. *C. albicans* is generally a commensal organism but may cause superficial mucosal diseases, oropharyngeal candidiasis (thrush), or vulvovaginal candidiasis (VVC) in immunocompromised individuals. Studies estimate that 75% of healthy women are also at risk of VVC. *Candida* sp. are recognized by multiple pattern recognition receptors (PRRs), but the Dectin-1/Syk pathway in particular has emerged as an important component of the host arsenal for *Candida* recognition and modulates various immune functions 6, 21, 22, 23, 24, 25. Deficiency in Dectin-1 is linked to the development of mucocutaneous infections in humans and highlights the essential nature of this pathway [26]. NLRP3 is the primary inflammasome activated by *C. albicans in vitro* and *in vivo*. Engagement of Dectin-1 and the downstream adaptor Syk provides the necessary priming and activation signals for the canonical NLRP3 inflammasome [24]. By contrast, certain *C. albicans* strains trigger the noncanonical caspase-8 inflammasome in dendritic cells (DCs) through the assembly of a CARD9–Bcl-10–MALT1 complex [6]. Intriguingly, this noncanonical pathway of inflammasome activation is triggered extracellularly by Dectin-1 ligation and *Candida* internalization is not necessary [6]. Overall, these studies suggest redundancy in pathways for inflammasome assembly through the same upstream sensor. In addition, *Candida* displays remarkable morphological plasticity by switching from the yeast to a filamentous hyphal form, a key feature enabling adherence and tissue invasion at mucosal surfaces. This alteration results in NLRP3 activation by exposing the Dectin-1 ligand, β-glucan, which is normally shielded from recognition by mannoproteins. Accordingly, both the yeast form and mutant strains lacking Egf1, a principal regulator of filamentation, are incompetent in NLRP3 activation 24, 27, 28. Highly polarized hyphae may also inflict physical damage on the host cell via rupture of phagosomal and cell membranes, thereby generating DAMPs for NLRP3 activation. However, these findings have been challenged by the discovery of mutant strains that fail to activate NLRP3 yet assemble normal hyphal filaments 29, 30, thereby signifying the involvement of additional, unrecognized microbial factors in NLRP3 activation.

Another important determinant of *Candida* pathogenesis, and inflammasome activation, is the extracellular proteolytic activity produced by a family of ten secreted aspartyl proteinases (Saps). Besides their plausible role in the evasion of host immunity, Saps also enable the fungus to adhere and invade host tissues. Family member Sap2 activates the NLRP3 inflammasome *in vitro* and *in vivo*, where vaginal inoculation of the purified full-length Sap2 resulted in local neutrophil influx and IL-1β accumulation in the vaginal fluid 31, 32. These measurements decreased on treatment with anti-Sap2 antibody or infection with mutant *Candida* Δ*Sap1*–*3* [32]. Whether Sap2's enzymatic activity is also required to trigger the inflammasome is unclear, as contradictory results exist *in vitro* and *in vivo*. It has been suggested that Sap2 activity serves only to hydrolyze the mucosal layer for efficient *Candida* invasion *in vivo* [31]. Any direct role for Sap2 enzymatic activity in inflammasome activation remains to be determined. In addition, these studies need further validation, as the yeast form of *Candida* produces Sap2 but is not an effective inflammasome activator 33, 34, 35, 36. Hypha-associated members Sap4, 5, and 6 are also expressed robustly during VVC 33, 34, 35, 36. In contrast to the previous study, intravaginal challenge with Δ*Sap5* mutant but not the triple knockout Δ*Sap1*–*3* resulted in reduced IL-1β secretion and polymorphonuclear leukocyte (PMN) flux [36]. These differences in results could be due to the different genetic background of animals used and/or differences in the preparation and dose of *Candida* infection. Although conflicting, these studies propose essential functions for Saps in inflammasome-mediated immunopathogenesis of VVC. However, more evidence is needed to identify the important Saps, which should enable improved understanding of the complex interplay between host and pathogen at the vaginal interface.

**Figure 1:**
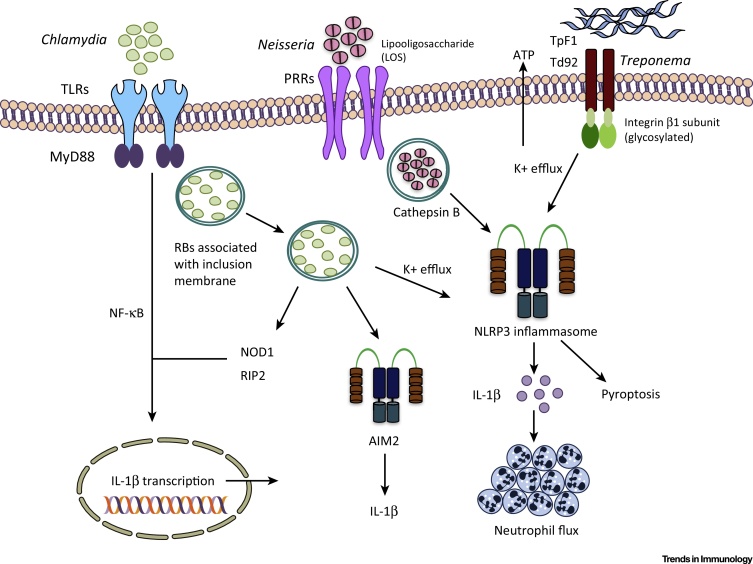
Inflammasome Activation Mechanisms during Bacterial Sexually Transmitted Infections (STIs). Activation of the NLRP3 inflammasome occurs through the ligation of various pattern recognition receptors (PRRs). *Chlamydia* is taken up in a vacuole known as an inclusion, where activity of the *Chlamydia* type III secretion system triggers the NLRP3 inflammasome. *Neisseria* releases membrane lipooligosaccharide, which has been suggested to activate the NLRP3 inflammasome through a cathepsin-B-dependent pathway. *Treponema* surface proteins TpF1 and Td92 activate the NLRP3 inflammasome dependent on K^+^ efflux. Since our understanding of inflammasomes and their activation mechanisms is incomplete in bacterial STIs, other mechanisms may also be involved. It is also unclear whether the same mechanisms are important *in vivo*.

*Neisseria gonorrhoeae*, the causative agent of gonorrhea, results in acute urethritis and cervicitis in males and females, respectively. *Neisseria* lipooligosaccharide (LOS), a modified form of LPS, has been suggested to activate the NLRP3 inflammasome and IL-1β secretion in a cathepsin-B-dependent manner [37] (Figure 1). However, invasion of the host cytosol by *N. gonorrhoeae*, or a role for additional gonococcal antigens as possible NLRP3 activators, cannot be completely excluded. Similar to gonorrhea LOS, *Treponema pallidum* TpF1, a bacterioferritin and a major virulence factor of this spirochete, also activates the NLRP3 inflammasome. *T. pallidum* causes syphilis, a sexually transmitted chronic inflammatory disorder that is characterized by mucocutaneous rash with enhanced vascular inflammation and angiogenesis [38]. TpF1 elicits pro-IL-1β production by monocytes, thus priming the inflammasome, and triggers the secretion of ATP, a known activator of NLRP3 [39]. Thus, TpF1 delivers both of the signals required for inflammasome activation (Figure 1). A related treponeme, *Treponema denticola*, implicated in human periodontal disease, also activates the NLRP3 inflammasome. Although rare, *T. denticola* may cause vaginitis and affect preterm delivery [40]. Interaction of Td92, a *T. denticola* surface protein, with monocyte membrane integrin α5β1 prompted ATP release and K^+^ efflux preceding NLRP3 activation [41]. Contrary to the requisite cytosolic presence of microbial ligands for NLRP3 trigger, activation by Td92 is independent of its internalization, and direct binding of recombinant Td92 to the glycosylated β1 subunit of integrin is mandatory for inflammasome activation (Figure 1).

## Positive and Negative Consequences of NLRP3 Inflammasome Activation

During infection, mice lacking *Nlrp3* display enhanced susceptibility to various infectious agents. By contrast, gain-of-function mutations in the *Nlrp3* gene lead to inflammatory diseases together known as cryopyrin-associated periodic syndromes (CAPSs). Thus, the role of the NLRP3 inflammasome is highly context dependent. In the case of sexually transmitted diseases, the importance of inflammasome activation *in vivo* is similarly context dependent and the use of appropriate models for the study of STIs is needed. *Chlamydia trachomatis* infection results in scarring of the ovaries and Fallopian tubes and is considered the leading cause of tubal infertility. Even when procreation is achieved, infection may result in ectopic pregnancy, preterm birth, and vertical transmission to the developing fetus 42, 43. Much of our understanding of the pathogenesis of and immune responses to *C. trachomatis* has developed through equivalent mouse models of *Chlamydia muridarum*, where the genital tract pathology is comparable to that in humans 44, 45. *Chlamydia* infection activates the NLRP3 inflammasome (Figure 1). Surprisingly, caspase-1 deficiency resulted in similar *C. muridarum* growth in the intravaginally infected mouse model, and the levels of shed live organisms were comparable at days 17 and 21 post-infection [46]. In terms of genital tract pathology, abolition of caspase-1 or IL-1 receptor (IL-1R) signaling reduced inflammatory damage in the oviducts 46, 47. Of note, the pathology does not appear to be affected in *Nlrp3*-deficient mice [47], suggesting that other inflammasomes may be involved. One hypothesis postulates that inflammasome-mediated pathology is due to rapid IL-1β-mediated PMN influx. Accordingly, abrogation of IL-1R signaling diminished pathology in the genital tissue and correlated with reduced PMN recruitment [47]. It is intriguing to consider that the infiltrated neutrophils can also supplement active IL-1β through cleavage of the precursor form by neutrophil proteases and thus exacerbate oviduct pathology during intravaginal challenge. Overall, these studies suggest that the inflammasome *per se* does not affect intravaginal *Chlamydia* colonization but augments detrimental pathology in the upper genital tract during the innate phase of infection.

Inflammasome activation also appears detrimental during VVC. Experiments in wild-type (WT) mice implicated NLRP3 activity as the source of increased PMN recruitment, increased production of alarmins, and elevated levels of IL-1β in vaginal lavage fluid during VVC. Consequently, infection in *Nlrp3*-deficient mice or treatment of WT mice with the NLRP3 inhibitor glyburide reduced *C. albicans* vaginitis without affecting microbial colonization [36].

Contrary to results described during genital infection, inflammasome activation during *C. pneumoniae* lung infection is critical for both elimination of the pathogen and protection from lung fibrosis 48, 49. Independent reports suggest that *C. pneumoniae* may also activate cytosolic sensors distinct from those activated by *C. muridarum or C. trachomatis* 50, 51. Similarly, mice lacking components of the NLRP3 inflammasome and upstream fungal recognition receptors are susceptible to disseminated candidiasis 24, 28, 52. In addition, deficiency in IL-1β, or loss of IL-1R signaling, promotes susceptibility due to the impact of this pathway on granulocyte influx and superoxide production [53]. Finally, administration of recombinant IL-18 protects against infection, and this occurs through the restoration of type 1 immunity 54, 55, 56. Overall, the results obtained from vaginal infection with either *Chlamydia* or *Candida* suggest that systemic infection or infection at distal sites with similar pathogens cannot be used to infer the roles of the inflammasome in the genital tract. Instead, appropriate pathogen strains and infection routes are essential to elucidate a clear picture of the function of inflammasomes in STIs.

Vaginal infection in a mouse model of HSV-2 demonstrated that *Il18*^−/−^ mice died sooner than WT mice and viral titers were higher in *Il18*^−/−^ mice on day 3 after infection [57]. However, following secondary challenge with HSV-2 in a memory recall experiment, *Il18*^−/−^ mice were fully protected, suggesting that IL-18 is not required for the development of appropriate immune memory [57]. Unfortunately, little else has been reported regarding the importance of the inflammasome for HSV-2. Increased inflammasome activation was also associated with increased protection from HSV-1 infection but in an ocular infection model [58]. A second report showed that *Nlrp3*^−/−^ mice are more susceptible to HSV-1 infection in an ocular infection model, but this was independent of inflammasome activation [59]. Indeed, the latter study reported that IL-1β levels were higher in *Nlrp3*^−/−^ mice following HSV-1 infection and noted that NLRP3 was localized to the nucleus. The authors hypothesized that NLRP3 has an inflammasome-independent function in immune regulation that helps to suppress deleterious inflammation in the ocular model of HSV-1 infection [59]. NLRP3 reportedly has inflammasome-independent roles 60, 61, 62, 63 but more research is needed to fully understand these potential functions. Furthermore, inflammasome activation may be important in ocular models, but whether this activity will be recapitulated during sexual transmission is unclear as activation of the inflammasome in human keratinocytes did not affect HSV-1 replication 64, 65 and inflammasome activation in vaginal models of HSV-1 have not been reported.

## Interplay between Distinct Inflammasomes

**Figure 2:**
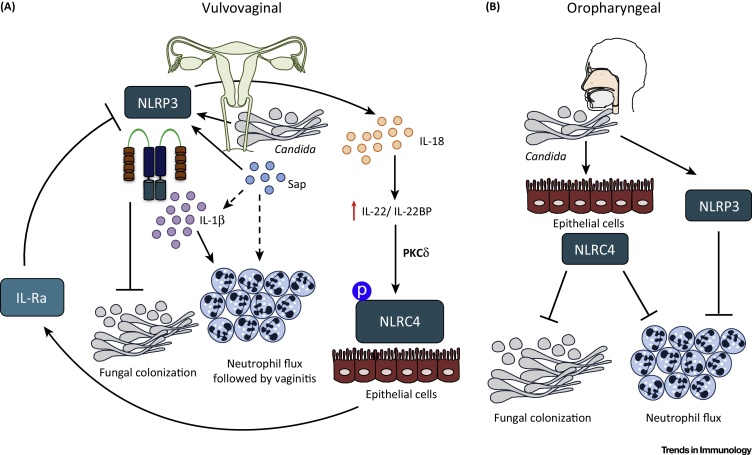
Key Figure: NLRP3 and NLRC4 Inflammasomes Mediate Distinct Host Immune Responses during *Candida albicans* Vaginal and Oral Infection

Infection with a pathogen can concurrently engage multiple inflammasome sensors 66, 67. A recent finding demonstrated the complex interplay of NLRP3 and NLRC4 during *C. albicans* infection in the vaginal tissue [68]. The expression of both of the inflammasome-activating sensors was augmented during VVC; however, NLRP3 expression peaked earlier in the vaginal tissue than the active phosphorylated form of NLRC4 (pNLRC4), which increased even further under *Nlrp3*-deficient conditions [68]. Further mechanistic studies associated NLRC4 activation, through an IL-22- and PKCδ-mediated pathway, with dampening exaggerated inflammation through the production of IL-1 receptor antagonist (IL-Ra) (Figure 2, Key Figure). Intriguingly, PKCδ is critical downstream of several Syk-coupled CLRs with roles in antifungal immunity, including Dectin-1, Dectin-2, and Mincle [69]. Accordingly, IL-22 administration *in vivo* dampened cytotoxic damage in the vaginas of infected mice. Conversely, treatment with an inhibitor of PKCδ decreased pNLRC4 expression and enhanced NLRP3-associated vaginitis [68]. These studies suggest that NLRC4 negatively regulates NLRP3 activity (Figure 2). Additionally, they suggest that sustained production of IL-1Ra by NLRC4 dampens NLRP3-mediated inflammation during VVC. Although VVC and oropharyngeal candidiasis involve similar inflammasomes, their activation seems to produce opposite results. During oropharyngeal candidiasis, akin to VVC, both NLRP3 and NLRC4 inflammasomes regulate IL-1β production. However, epithelial *Nlrc4* deficiency, more than *Nlrp3*, resulted in significantly enhanced *Candida* buccal load throughout the 21-day infection period and increased inflammatory cell recruitment in the tongue epithelium despite the presence of erosive lesions and hyphae [70] (Figure 2). Notably, *Nlrp3* deficiency resulted in only slightly elevated oral colonization and gross clinical score [70]. Thus, although both oral and vaginal infections are clinical manifestations of mucosal infection, inflammasome activation and enhanced PMN infiltration leads to contrasting results at the two sites.

## Inflammasome-Induced Pyroptosis in STIs

**Figure 3:**
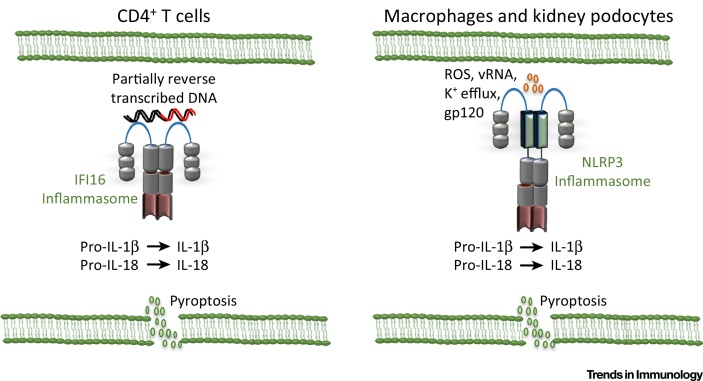
Pyroptosis during HIV Infection Contributes to Disease. During HIV infection, different inflammasomes are activated in different cell types and in response to different stimuli. However, in all cell types inflammasome activation causes maturation of IL-1β and the inflammatory form of cell death, pyroptosis. Pyroptosis in CD4^+^ T cells contributes to immunodeficiency by further depleting CD4^+^ T cell numbers, whereas pyroptosis of podocytes in the kidney results in HIV-associated nephropathy.

HIV is one of the most concerning worldwide pandemics, with approximately 37 million people infected with a virus that causes lifelong morbidity and eventual mortality [71]. HIV infection results in the activation of both the NLRP3 and IFI16 inflammasomes (Figure 3). In monocytes, the NLRP3 inflammasome is activated in response to HIV infection as a result of TLR8-mediated priming and reactive oxygen species (ROS) production 72, 73, 74. However, IFI16 appears to be the predominant inflammasome activated in CD4^+^ T cells and may lead to AIDS progression [75]. Activation of inflammasomes results in a programmed cell death termed pyroptosis. Pyroptosis of the infected cells results in destruction of the pathogen replicative niche. However, because of the inherently inflammatory nature of this form of cell death, it may promote tissue damage. IFI16 activation and pyroptosis in response to HIV infection results in the depletion of resting CD4^+^ T cells, which further exacerbates immunodeficiency 75, 76, 77, 78. Direct infection of CD4^+^ T cells does not appear to result in pyroptosis. Instead, cell-to-cell transmission though the virus synapse results in abortive infection of resting CD4^+^ T cells and the accumulation of reverse-transcribed HIV genomes in the cell (Figure 3). These DNA molecules are then sensed by the IFI16 inflammasome resulting in pyroptosis [79]. Interestingly, cell-to-cell spread occurs most efficiently in the lymph node environment and not in blood-circulating CD4^+^ T cells [76]. In the kidneys, HIV-associated nephropathy results from the loss of podocytes. Recent research suggests that NLRP3 inflammasome activation in the kidneys during HIV infection causes pyroptotic cell death of podocytes and contributes to kidney damage (Figure 3) [80]. Furthermore, inhibition of ROS or the NLRP3 inflammasome resulted in improved podocyte survival in the Tg26 transgenic mouse model of HIV infection [80]. Overall, inflammasome activation by HIV appears to do more harm than good, and it will be of interest to determine the therapeutic potential of inflammasome inhibition.

Induction of pyroptosis during *Chlamydia* infection causes injury to the upper genital tract resulting in degeneration of oviduct epithelia, swollen oviducts, and widespread necrosis of the endometrium [81]. Inflammasome assembly was demonstrated to induce pyroptosis in antigen-presenting DCs in an IL-10-dependent manner [81]. Consequently, IL-10 abolition reduced inflammasome activation and limited necrosis in the endometrium. Additionally, *Chlamydia*-infected *Il10*-deficient mice had 100% fertility but *Chlamydia*-infected WT mice suffered significant fertility impairment. However, mechanistic pathways coupling IL-10 to NLRP3 in DCs remain unclear. Furthermore, these results appear contradictory to the emerging role of IL-10 as a negative regulator of inflammasome signaling 82, 83. Nonetheless, the inflammasome-dependent pathology encountered by the host seems to be restricted to primary infection, as pathology encountered during recurrent infections is propagated by adaptive immunity [46].

## Polymorphism or Expression Changes in Inflammasome-Coding Genes

The role of inflammasomes during VVC is also corroborated by studies in humans where polymorphism in the gene encoding *Nlrp3* is associated with increased incidence of recurrent VVC (RVVC), which is characterized by at least three episodes of infection per year 84, 85. One study measured inflammasome-dependent cytokine production at the mucosal surface and observed enhanced IL-1β levels in the vaginal fluid of RVVC patients compared with healthy controls. Intriguingly, RVVC patients bearing the risk allele demonstrated even higher levels of IL-1β production [84]. In agreement, IL-1Ra levels were lower in recurrent VVC patients. Additionally, IL-18 levels were unaltered in the vaginal fluid of patients bearing the risk allele [84]. These studies thus argue that genetic variations in the *Nlrp3* gene may influence the progression of VVC and identify IL-1β as a therapeutic target in the management of RVVC.

Several targeted genetic association studies have found that certain alleles of IL-1β, IL-18, NLRP3, and NLRP1 are associated with resistance or susceptibility to more severe human papillomavirus (HPV) outcomes such as cervical cancer 86, 87. Two other studies reported downregulation of the expression of IL-1β and other inflammasome-related genes in patients who are HPV infected or have developed cervical cancer 88, 89. Furthermore, elevated IFI16 and AIM2 expression is associated with HPV infection and HPV-associated cancer development 88, 90, 91. AIM2 may respond to HPV infection of human keratinocytes by detecting viral DNA in the cytoplasm. However, this finding was not based on a natural infection. Instead, viral genomic DNA was transfected into keratinocytes; thus, the role of AIM2 during natural HPV infection is unknown [92]. Finally, inflammasome activation during HIV infection results in negative immunopathologic effects as described above. It is thus interesting to note that polymorphisms in NLRP3 and IL-1β are found more commonly in HIV-positive individuals than in uninfected individuals 93, 94. Although the functional consequences of these polymorphisms are unknown, it will be of interest to determine whether they enhance or inhibit inflammasome activation, potentially facilitating pyroptotic cell death and leading to disease progression or resulting in impaired immunity with increased disease susceptibility.

## Concluding Remarks

The contributions of inflammasomes during STIs are only beginning to be understood. Recent studies have depicted the significance of inflammasomes *in vitro* in response to sexually transmitted pathogens. However, few *in vivo* studies have been conducted and this remains challenging because of the topology of infection site and lack of appropriate animal models that faithfully recapitulate the infection. Nevertheless, a few well-controlled studies employing intravaginal challenge models of *Candida* and *Chlamydia* have depicted detrimental roles of inflammasomes in the genital tract, in contrast to results observed *in vitro* and in systemic models of infection. These significant differences highlight the importance of performing discovery-based experiments using specific models instead of drawing conclusions solely from related studies. These studies also illustrated activation of distinct inflammasomes in hematopoietic and stromal compartments, thereby highlighting the need to develop tissue-specific models and conditional knockouts that accurately measure the contribution of each inflammasome type. Nevertheless, whether the detrimental role of inflammasomes in the genital tract extends to other STIs remains to be examined. Regardless of the infection, improved models of STIs are needed to better understand the role of inflammasomes in STIs. Especially, there is a need for the development of models that recapitulate the initial sexual transmission of the infection and allow examination of the initial immune responses that are involved in facilitating or preventing disease transmission. There is little doubt that inflammasomes are activated during STIs. The major question is which inflammasome types are important in the skin and mucosal tissues? Also, what are the precise pathways that pair each STI to a specific inflammasome? These and other questions remain enigmatic (see Outstanding Questions), but by understanding the nature of protection and damage mediated by inflammasomes these studies will further advance our knowledge and are essential for reproductive health. Finally, an improved grasp of the role of inflammasomes in the genital tract may translate into new therapeutic opportunities to reduce morbidity and mortality due to STIs.

Outstanding Questions Which inflammasome types are critical in the genital tract? What are the molecular pathways that activate inflammasomes in the genital mucosa? We have increased understanding of inflammasome signaling in hematopoietic cells but our knowledge of immune receptors and inflammasome activation mechanisms in the mucosal epithelium is rather limited.

What are the roles of the noncanonical NLRP3-dependent and NLRP3-independent inflammasomes? Recent reports have suggested key roles for inflammasomes other than NLRP3 during a wide variety of infections. However, their roles in STIs remain ambiguous. For example, activation of the AIM2 inflammasome by *Candida* sp. was recently described in macrophages. Does AIM2 also influence progression of vulvovaginal candidiasis?

What is the role of autophagy during STIs? Both autophagy of pathogens (xenophagy) and autophagic degradation of inflammasomes and precursor IL-1β by macroautophagy are now considered important mechanisms contributing to infection outcome. However, these mechanisms have not been characterized in STIs.

Which host pathways function as rheostats between pathogen elimination and exaggerated inflammatory responses? Since inflammasomes have both beneficial and detrimental roles, there is a need to identify targets that can specifically activate or dampen inflammasome activity. Knowledge in this area can help us develop appropriate therapeutic interventions.

What are the functional consequences of inflammasome gene polymorphisms in the human population? Numerous studies report that specific alleles of genes encoding inflammasome components are associated with increased propensity to infection or severe disease, but the functional consequences of these alleles and how they predispose patients to disease are unknown.

## Figures and Tables

**Table 1:** Ligands of Inflammasome Sensors in STIs.

PRR	STI PAMP/DAMP	Pathogen	Refs
NLRP3	Secreted aspartyl proteinases, ROS, K^+^ efflux, and other cellular damage signals	HPV	[87]
		HSV	58, 64
		HIV	73, 80, 93, 94, 95, 96
		*Chlamydia trachomatis*, *Chlamydia muridarum*	46, 47, 81
		*Neisseria gonorrhoeae*	[37]
		*Treponema pallidum*	39, 41
		*Candida albicans*	6, 24, 27, 28, 31, 32, 36, 68
NLRC4	Flagellin, T3SS components, or unknown fungal ligands	*C. albicans*	36, 68
AIM2	Cytoplasmic dsDNA	HPV	[92]
		HSV	[65]
		*Chlamydia*	[97]
IFI16	Nuclear dsDNA	HSV	64, 98, 99
		HIV	75, 76, 77, 79

## Box 1 Innate Immunity in the Genital Tract

The reproductive system, of both males and females, is integral to the inner mucosal lining of the human body. Similar to the mucosal tissues of the intestine and lungs, the reproductive tract is able to mount comprehensive immune responses to pathogens. In addition to invading microbes, the female lower genital tract also contains a unique microflora. Thus, the cervical and vaginal epithelial cells are in frequent contact with microorganisms while also occasionally facing antigenic and inflammatory stimuli during intercourse [100]. Therefore, the genital tract is fortified by both innate and adaptive immune responses. However, in keeping with its paramount role in reproduction, distinct adaptations have evolved.

The female genital mucosa comprises a single layer of tightly packed columnar epithelial cells in the upper reproductive tract. By contrast, the lower reproductive tract is made of multiple layers of stratified squamous epithelium. The outer epithelial layer is further coated on the luminal side by a thick mucus layer comprising glycosylated proteins known as mucins. Together, epithelial cells and the mucus layer form both physical and immunological barriers to prevent infection and transmission of STIs; nonetheless, invasion does occur. Once these barriers are breached by pathogens, epithelial cells are infected followed by the activation of macrophages and DCs. These tissue-resident macrophages and DCs endeavor to eliminate the infectious agent by phagocytosis while augmenting cytokine and chemokine production to expand cellular recruitment. Moreover, activated DCs serve to bridge innate and adaptive immunity by migrating into draining lymph nodes where they prime antigen-specific T cells and initiate B cell responses 100, 101, 102, 103.

In addition to the expression of inflammasome sensors (which are discussed in detail below), several PRRs are expressed in the epithelial cells of the reproductive tract. The NLRs NOD1 and NOD2 and their adaptor protein RIP2 are expressed in the human endometrium 104, 105. In addition, TLRs including TLR1, 2, 3, and 6 are found in the genital mucosal epithelium [101]. Some of the TLRs have a unique site-specific expression pattern in the reproductive tract tissue 105, 106, 107, 108, 109, 110. For example, TLR4 is mainly expressed in the upper genital tract and the expression gradually decreases in the lower genital tract 105, 108, 110. It has been suggested that this gradient of TLR distribution is likely to reflect the immunologic tolerance of the lower genital tract to commensal organisms while maintaining a firm intolerance to commensals and pathogens in the upper genital tract, which is important for reproduction. Thus, commensal flora, but also reproductive hormones and locally available cues, shape the immune responses and differentiation of cells in the upper and lower genital tract. The role of these factors as well as non-inflammasome PRRs in the reproductive system are discussed in depth elsewhere 101, 103, 111, 112. However, many non-inflammasome PRRs have essential roles in priming inflammasomes, as discussed above and thus a discussion of their importance relative to inflammasome activation during STIs is included.
